# De novo transcriptome sequencing of black pepper (*Piper nigrum* L.) and an analysis of genes involved in phenylpropanoid metabolism in response to *Phytophthora capsici*

**DOI:** 10.1186/s12864-016-3155-7

**Published:** 2016-10-21

**Authors:** Chaoyun Hao, Zhiqiang Xia, Rui Fan, Lehe Tan, Lisong Hu, Baoduo Wu, Huasong Wu

**Affiliations:** 1 Spice and Beverage Research Institute, Chinese Academy of Tropical Agricultural Sciences (CATAS), Wanning, Hainan 571533 China; 2 Institute of Tropical Biosciences and Biotechnology, Chinese Academy of Tropical Agricultural Sciences (CATAS), Haikou, 571101 China; 3 Key Laboratory of Genetic Resources Utilization of Spice and Beverage Crops, Ministry of Agriculture, Wanning, Hainan 571533 China; 4 Hainan Provincial Key Laboratory of Genetic Improvement and Quality Regulation for Tropical Spice and Beverage Crops, Wanning, Hainan 571533 China

**Keywords:** Black pepper, Resistance, Mechanism, Illumina sequencing, Phenylpropanoids

## Abstract

**Background:**

*Piper nigrum* L., or “black pepper”, is an economically important spice crop in tropical regions. Black pepper production is markedly affected by foot rot disease caused by *Phytophthora capsici*, and genetic improvement of black pepper is essential for combating foot rot diseases. However, little is known about the mechanism of anti- *P. capsici* in black pepper. The molecular mechanisms underlying foot rot susceptibility were studied by comparing transcriptome analysis between resistant (*Piper flaviflorum*) and susceptible (*Piper nigrum* cv. *Reyin-*1) black pepper species.

**Results:**

116,432 unigenes were acquired from six libraries (three replicates of resistant and susceptible black pepper samples), which were integrated by applying BLAST similarity searches and noted by adopting Kyoto Encyclopaedia of Genes and Gene Ontology (GO) genome orthology identifiers. The reference transcriptome was mapped using two sets of digital gene expression data. Using GO enrichment analysis for the differentially expressed genes, the majority of the genes associated with the phenylpropanoid biosynthesis pathway were identified in *P. flaviflorum*. In addition, the expression of genes revealed that after susceptible and resistant species were inoculated with *P. capsici*, the majority of genes incorporated in the phenylpropanoid metabolism pathway were up-regulated in both species. Among various treatments and organs, all the genes were up-regulated to a relatively high degree in resistant species. Phenylalanine ammonia lyase and peroxidase enzyme activity increased in susceptible and resistant species after inoculation with *P. capsici*, and the resistant species increased faster. The resistant plants retain their vascular structure in lignin revealed by histochemical analysis.

**Conclusions:**

Our data provide critical information regarding target genes and a technological basis for future studies of black pepper genetic improvements, including transgenic breeding.

**Electronic supplementary material:**

The online version of this article (doi:10.1186/s12864-016-3155-7) contains supplementary material, which is available to authorized users.

## Background

*Piper nigrum* is an important member of the family Piperaceae, which is chiefly cultivated as a major cash crop more than 30 tropical countries of the world, such as Vietnam, India, Malaysia, Indonesia, China, and Brazil [1, 2]. The species, often called “black pepper”, is considered as the “king of spice” due to its global trade and widespread dietary, medicinal, and preservative uses [2–4]. Black pepper is widely cultivated as an important spice, with yields reaching 461,452 tons and profits exceeding 10 billion US dollars [5]. As the most significant black pepper disease, foot rot, caused by *Phytophthora capsici* Leonian which infects roots, stems, leaves and fruit throughout the entire process of plant growth, is observed in almost all the plantation [6, 7]. The epidemic development of black pepper foot rot is associated with cultivation techniques, soil moisture, soil fertility, environmental conditions and drainage [6]. To date, researchers have not developed an efficient controlling method for this disease because it is a soil-borne vascular disease [8, 9]. Attempts to improve black pepper resistance to *P. capsici* through conventional breeding have been unsuccessful [8]; anti-*P. capsici* black pepper varieties have not been bred.

Given the scientific and economic value of the genus *Piper*, many researchers have focused on this genus in past decades. Many of these studies have been dedicated to understanding the intrageneric molecular phylogeny, the genetic relationships and fingerprints, the genetic distribution patterns of some endemic species, and so on [3]; however, little is known about the genetics of black pepper [10]. Sheila et al. [4] had built the first dataset of sequences in *P.nigrum*. However, few studies have addressed the molecular mechanisms of black pepper foot rot caused by *P. capsici*. New developments in bioinformatics technologies and molecular biology have facilitated the understanding of the molecular mechanism of foot rot and have enhanced plant resistance to disease by means of plant genetic engineering. Through complicated transduction, perception and signal exchange, a range of defence mechanisms are adopted by plants to avoid pathogen attacks [11, 12].

Using next-generation sequencing (NGS) technology, transcriptome sequencing offers much data with tremendous coverage and more sequence depth. RNA-Seq can help to better understand significant variations in metabolic processes, which is beneficial for gene discovery, comparative transcriptomics and evolutionary genomics [13, 14]. At present, discovering and identifying the genes were used by RNA-Seq, which are involved in the biosynthesis of secondary metabolites, including cellulose and lignin in Chinese fir and cotton [15]; the carotenoid in *Momordica cochinchinensis* [16]; flavonoid, theanine and caffeine [17]; flavonoid in safflower [18]; the active ingredients in *Salvia miltiorrhiza* [19]; and capsaicinoid in red pepper (*Capsicum annuum*) [20]. Various defending approaches are used by plants for protecting themselves from being harmed by pathogen through complicated perceiving, transducing as well as exchanging signals [21]. In plants, diversified metabolites are derived from phenylpropanoid pathway which exerts an essential role in biosynthesizing lignin and acts as the origin of synthesizing various other products including lignans, coumarins as well as flavonoids. Phenylpropanoids that are originated from phenylalanine carbon bone belong to a group of diversified metabolites which participate in structural supporting, defending as well as surviving [22]. It was proposed previously that in affected resistant tomato roots, the production of hydrogen peroxide was enhanced and the activity of phenylalanine-ammonia lyase (PAL) and peroxidase (POD) as well as the synthesis of lignin was increased [23]. Studies on the POD activity of different resistance cultivars of red pepper, cucumber and tobacco demonstrated that the increase in POD activity was higher in the resistant cultivar after infection than in the susceptible cultivar [24, 25]. The expression profiles of genes in plants during defending could be investigated by means of recently developed deep sequencing technology in a systematic way.

To get an overall comprehensive characterization of root transcriptome from resistant (*P. flaviflorum*) and susceptible (*P. nigrum cv. Reyin-1*), the Illumina HiSeq™ 2000 platform was used to identify the genes and pathway that involved in resistant plants. On the basis of global gene expression, enzyme activity and histochemical analysis, the phenylpropanoid metabolism related to black pepper defense response to *P.capsici* was detected*.* The results generated in this study provided fundamental information to the control of plant development and disease resistance in black pepper.

## Results

**Fig. 1:**
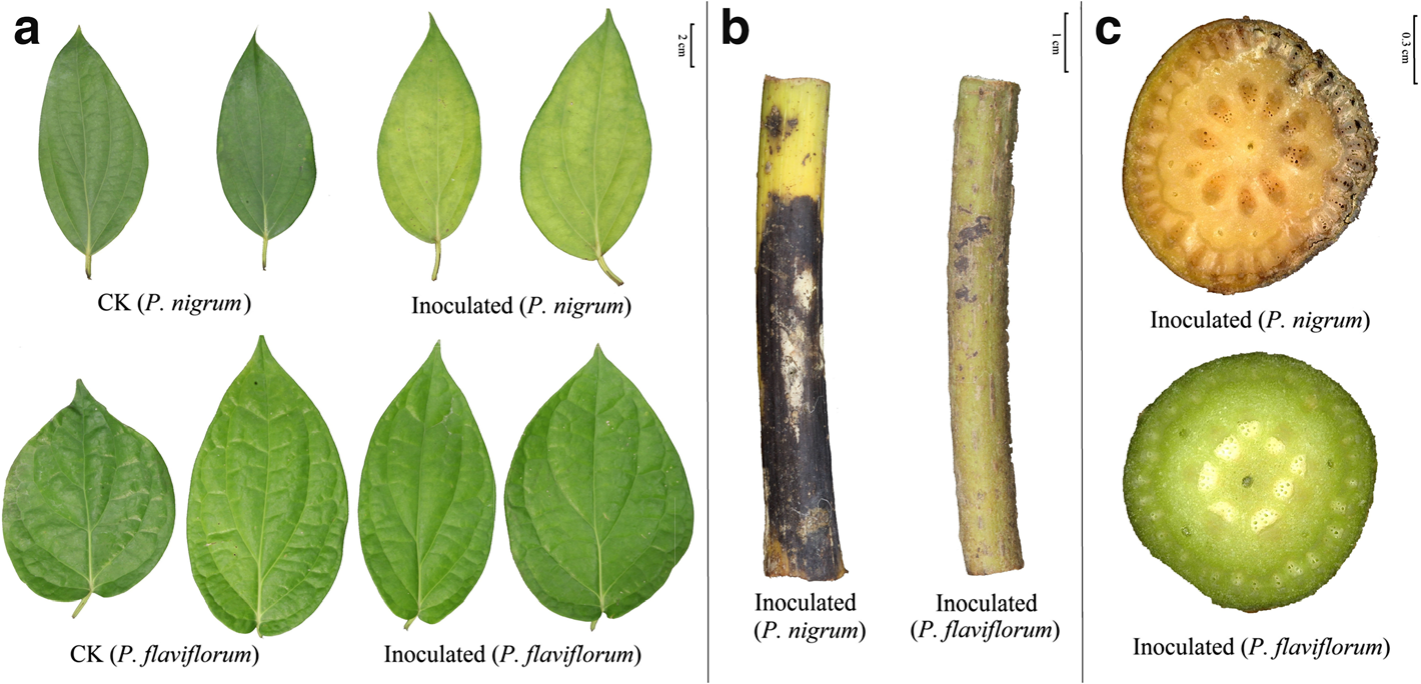
Disease symptoms on both resistant and susceptible species after *P. capsici* inoculation. **a** Leaves; **b** Stems; **c** Cross-sections of stems

Resistant (*P. flaviflorum*) and susceptible (*P. nigrum cv. Reyin-*1) of black pepper species were inoculated against foot rot disease caused by *P. capsici* using pin-pricking method*.* After 7 days, leaf chlorosis, stem browning and split open stems were exhibited in the susceptible species. Nevertheless, no external lesions were observed in plants with resistance after which were inoculated by pathogen under the condition of greenhouse (Fig. 1).

### Sequencing and de novo assembly

**Fig. 2:**
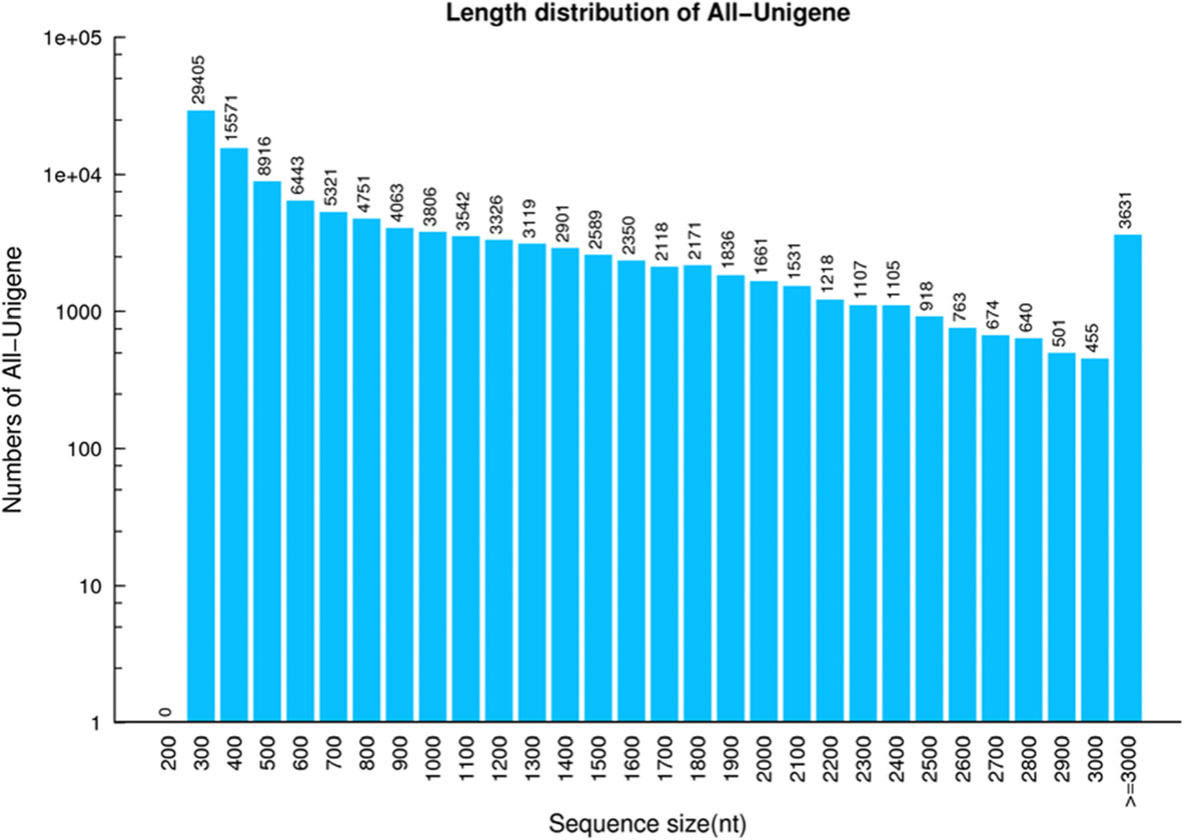
Sequence-length distribution of the transcripts assembled from the Illumina reads.

Six transcriptome libraries by using an Illumina HiSeq™ 2000 platform for two black pepper species (*P. flaviflorum* and *P. nigrum*), three biological replicates were constructed and sequenced, which were designated *P. flaviflorum*-1, *P. flaviflorum*-2*, P. flaviflorum*-3, *P. nigrum*-1 *P. nigrum*-2 and *P. nigrum*-3. Totally 16.28 M paired raw reads and six libraries with the Q20 values ranged from 98.17 to 99.33 % were produced in this study (Table 1). The reads with low quality or adaptors were removed and 49.66 M, 49.27 M as well as 48.51 M clean reads were generated for *P. nigrum*, while 49.46 M, 49.54 M and 48.49 M were generated for *P. flaviflorum* respectively. Subsequently, de novo assembly was conducted on these reads to obtain 363,233 unique transcripts in total corresponding to 116,432 genes with a GC content of 45.05 % and a N50 value of 1496. At least one half of total lengths of contigs including which had length of 1496 bp were represented. The properties of the assembly including the mean length of contigs are shown in Table 1. The proportion of sequences with matches in the non-redundant databases was greater among the longer assembled sequences, especially, most of the transcripts were between 201 bp and 400 bp in length (Fig. 2).

**Table 1:** Overview of transcriptome sequencing and de novo assemble results

	*P. nigrum*-1	*P. nigrum*-2	*P. nigrum*-3	*P. flaviflorum*-1	*P. flaviflorum*-2	*P. flaviflorum*-3
Raw reads (Mb)	49.26	54.44	54.44	49.26	54.44	54.44
Q20 percentage	99.33	98.24	98.30	99.33	98.23	98.17
Clean reads (Mb)	48.51	49.27	49.66	48.49	49.54	49.46
unigenes	62,206	55,880	54,681	61,187	64,183	65,096
Percent GC	45.23	45.57	45.48	45.27	45.03	45.58
Contig N50	1,131	1,065	1,069	1,301	1,091	1,026

### Annotation

**Fig. 3:**
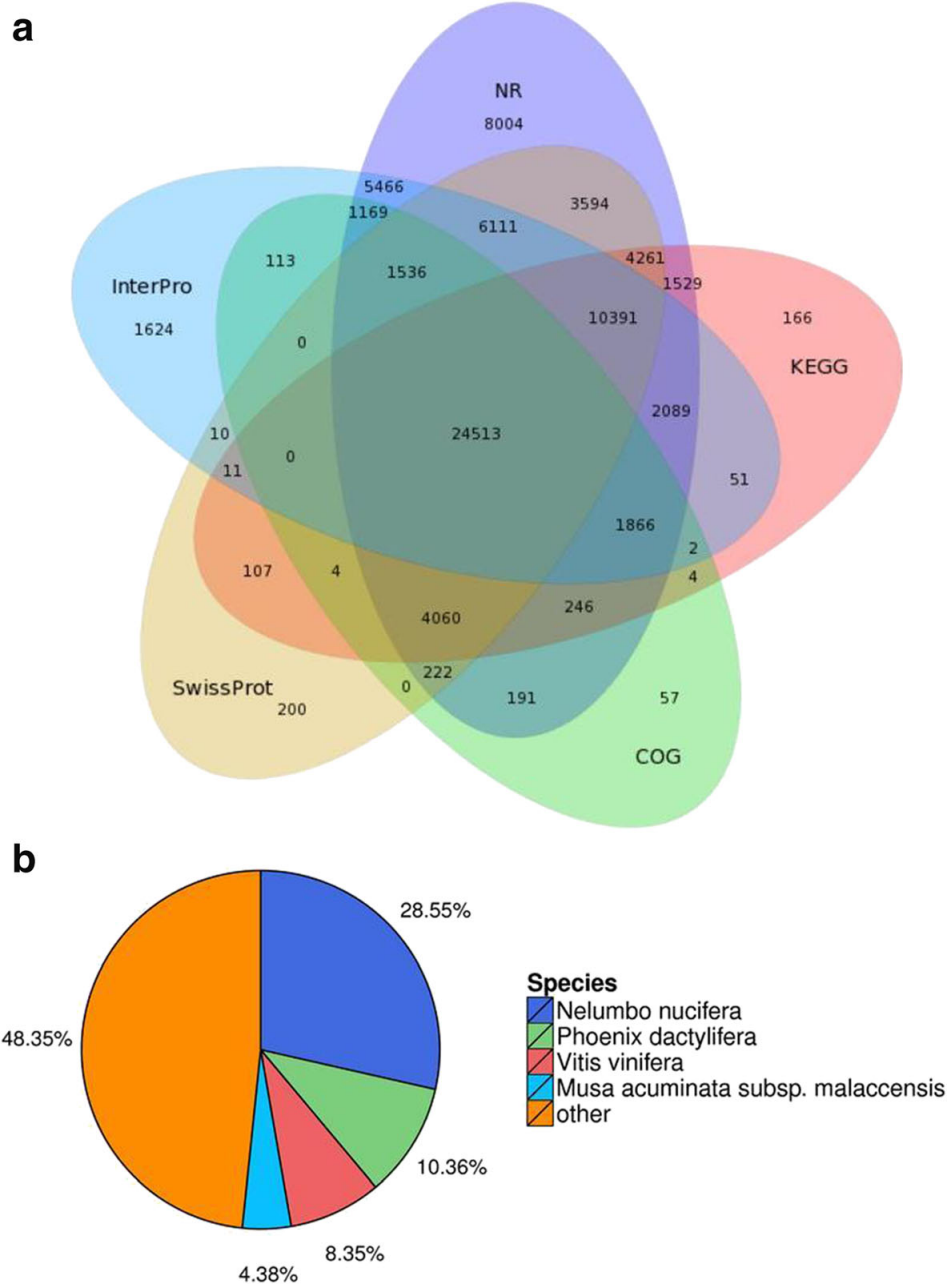
Results summary for the sequence homology search against the NCBI NR database. **a** Venn diagram of transcripts detected in NR, KEGG, interpro, swissprot and COG. **b** A species-based distribution of the BLASTX matches of the sequences. We used all of the plant proteins in the NCBI NR database to perform the homology search, and we selected the closest match for each sequence for the analysis

BlastX was used to search these sequences in NCBI NR database and threshold E-value was set as 10^−5^ in annotation. In total, of the 116,432 unigenes, 75,248 (64.63 %) unigenes showed significant similarity in the NR database. In the other four database (SwissProt, GO, KEGG, and COG), 55,020 (47.26 %), 33,127(28.45 %), 49,300(42.34 %), 33,983(29.19 %) were successfully aligned to known proteins. These assembled transcripts were mapped to a few public databases, the amount of transcripts which were aligned to each database are shown in Fig. 3a. Overall, 24,513 unigenes were annotated in five databases. In a further analysis of the matching sequences, we found that *Nelumbo nucifera* was ranked first, with 28.55 % of the sequences, followed by *Phoenix dactylifera* and *Vitis vinifera*; 48.35 % of the sequences showed matches with sequences from other species (Fig. 3b).

### Gene ontology (GO) and Kyoto encyclopaedia of genes and genomes (KEGG) classification

The function annotation was conducted on the basis of homology. BlastX was used to search all unique genes in NCBI NR protein database and 33,127 sequences were classified into 57 functional groups on the basis of homological sequences. GO classifications were divided into three major categories: biological process, cellular component and molecular function; “catalytic activity”, “metabolic process” and “cellular process” terms were dominant in each of these categories, respectively. We also noticed a high percentage of genes from the “cell part”, “cell” and “binding” categories and only a few genes from the “behavior” and “translation regulator activity”.

KEGG is a classification method based on pathways which could be used to profile the functions of genes. To identify biochemical pathways, 49,300 genes were mapped to 128 KEGG pathways. In particular, several important secondary metabolite biosynthetic pathways, including the phenylpropanoid, flavonoid and carotenoid biosynthetic pathways, as well as several signalling pathways, were represented.

### Differentially expressed genes (DEGs) in the pepper transcriptomes

**Fig. 4:**
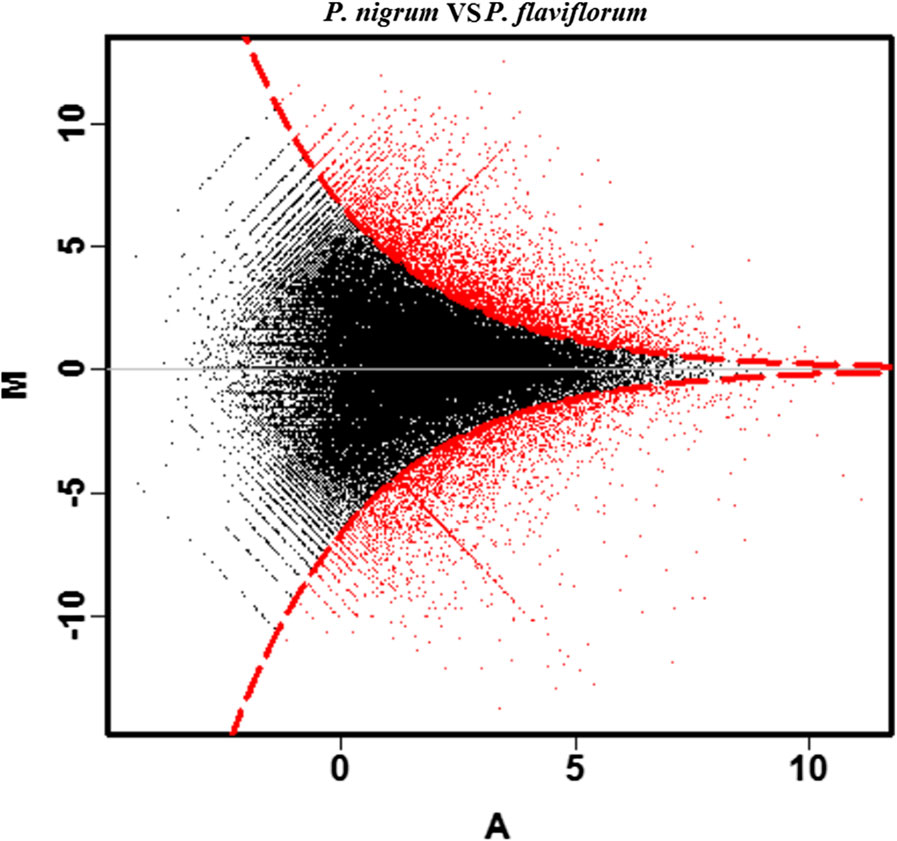
Changes in the transcript abundance levels between *P. nigrum* (susceptible) and *P. flaviflorum* (resistant). (M) Log_2_ (*P. nigrum/P. flaviflorum*); (A) Mean FPKM. The red dots indicate that the genes differed significantly in expression compared with the control, with Log_2_FPKM approaching a 0.05 *P*-value

**Fig. 5:**
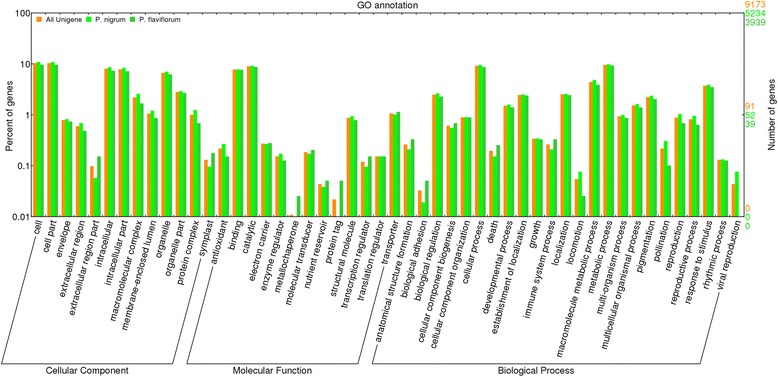
Histogram of GO (gene ontology) classifications of assembled unigenes and up- and down-regulated unigenes of black pepper. The results are summarized in the following three main categories: (1) biological process, (2) cellular component and (3) molecular function. The left y-axis indicates the percentage of the specific category of genes in the main category. The right y-axis indicates the number of genes in a specific category

The Illumina reads were mapped onto the combined transcriptome database to investigate the DEGs between the two species. The gene expression profiles of *P. flaviflorum* and *P. nigrum* were compared, and the expression of 3,450 DEGs was found between these species. The standards of this analysis included: (i) The copy numbers of tags in two samples were not lower than ten, (ii) The absolute value of fold change (log _2_ ratio) between two samples was not lower than two, and (iii) The max *P* value of differential expression between these two samples and the max *F*-distribution ratio of these results were 0.05. Higher expression levels were observed in 1,953 DEGs in *P. flaviflorum* (Fig. 4, Additional file 1: Data S1). The distributing patterns of up-regulated and down-regulated DEGs exhibited similarity (Fig. 5). Many of the DEGs were mapped to pathways that were essential for plant growth and development. The most frequently represented pathways were primarily involved in secondary metabolism. The cluster for “phenylpropanoid biosynthesis [PATH: ko00940]” represented the largest group, and most of genes were up-regulated in *P. flaviflorum* compared with *P. nigrum*. GO analysis of these genes revealed enrichment in the genes involved in specific metabolic pathways in the susceptible and resistant species. The resistant species showed enrichment in genes involved in “phenylpropanoid metabolic process” and “cellular amino acid derivative metabolic process”. In contrast, genes involved in “toxin metabolic process” and “cellular catabolic process” was enriched in the susceptible species (Additional file 2: Figure S1).

Arranging molecular interactions as networks and describing information flow within networks are primary targets of systems biology. To estimate the activation-inhibition relationships, a computational framework was presented to facilitate to the combination of protein-protein interaction (PPI) networks and genetic screens (Additional file 3: Figure S2). We derived the PPI database from Arabidopsis; this database can be used to find all of the genes in the phenylpropanoid pathway and includes their secondary network-related genes. We identified cinnamyl alcohol dehydrogenase (*CAD*), pathogenesis-related gene, ATCAX5, heat shock protein 70 and other genes in the resistant species.

### Analysis of phenylpropanoid biosynthesis pathway genes

The transcripts of “phenylpropanoid biosynthesizing pathway” were analysed in this study since it was the most important KEGG pathway among generally up-regulated transcripts. The identification of sequences originating from the phenylpropanoid biosynthesis pathway genes was performed using the *P. flaviflorum* NGS database. The phenylpropanoid biosynthesis and the identified genes incorporated in this pathway were summarized from six library; enzymes that play a role in the core phenylpropanoid pathway are presented, such as 4-coumarate: CoA ligase (*4CL*), *PAL* and cinnamoyl-CoA reductase (*CCR*). Additionally, hydroxycinnamoyl transferase (*HCT*) and cinnamate 4-hydroxylase (*C4H*) up-regulation occurred among the majority of identified genes included in the lignin biosynthesis pathway (Table 2, Additional file 4: Data S2). In general, the results revealed that phenylpropanoid pathway activation occurred in the resistant black pepper species.

**Table 2:** Unigenes matched to known phenylpropanoids.lignin biosynthesis factors

Gene name	Gene ID	ID	*P. flaviflorum*-1	*P. flaviflorum*-2	*P. flaviflorum*-3	*P. nigrum*-1	*P. nigrum*-2	*P. nigrum*-3	Log 2
4CL	CL1237.contig11.ALL	At1g51680	102.29	91.93	76.73	0.11	0.05	0.39	9.833996
HCT	CL1656 .contig4.ALL	At5g48930	0.61	0.1	0.16	91.92	50.1	40.05	−7.26238
CAD	CL1837.contig3.ALL	At2g21730	84.99	74.45	59.41	26.64	28.62	41.55	1.646749
CCR	CL215.contig4.ALL	At1g15950	235.92	0	0	0	0	0.21	8.855204
C4H	CL300.contig10.ALL	At2g30490	1.36	0.31	0.32	3.07	3.9	8.35	−1.17463
PAL	CL4018.contig4.ALL	At2g37040	14.15	21.16	19.48	3.06	1.59	1.05	2.209198

### qPCR analysis of genes

**Fig. 6:**
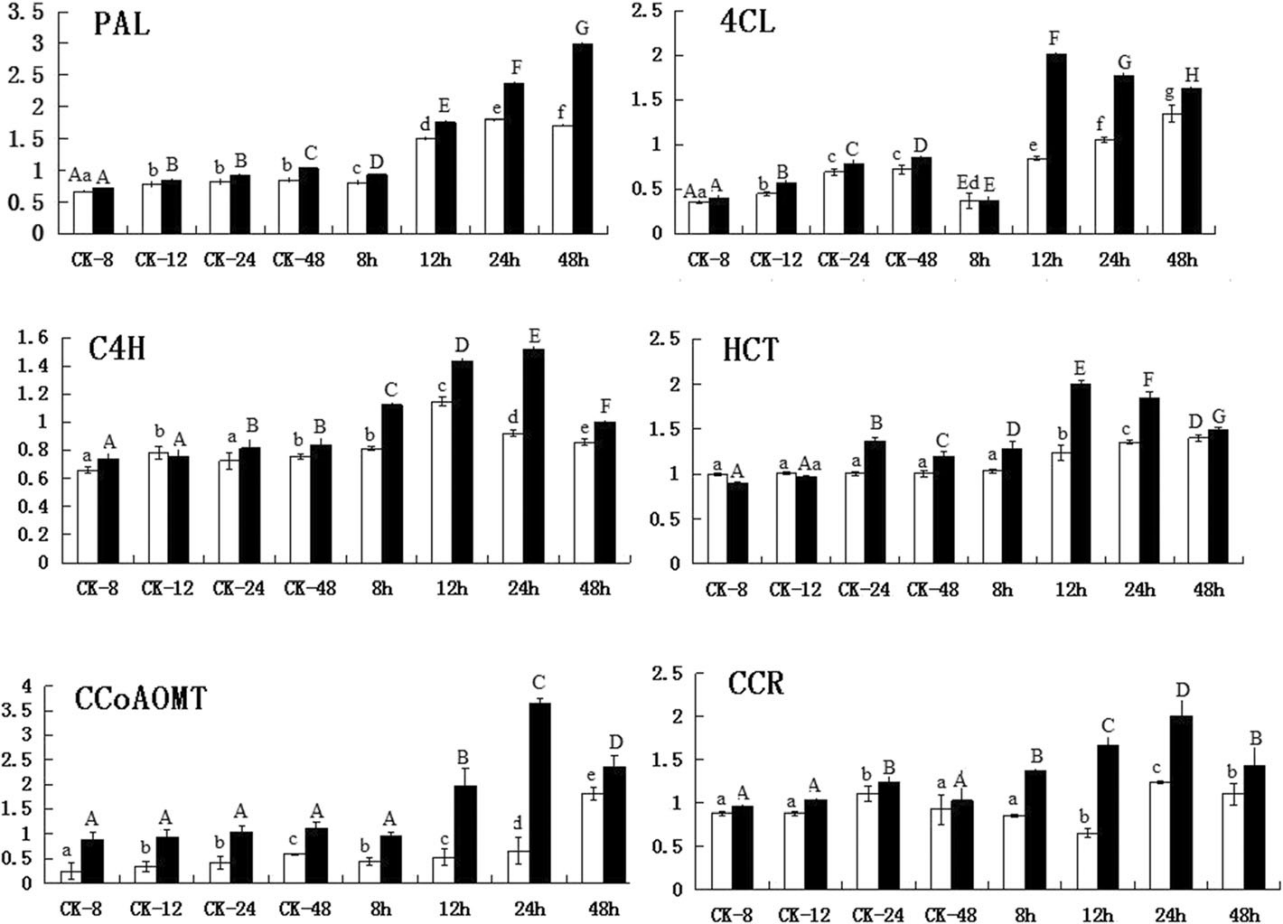
Detailed expression profiles of the genes in the major lignin biosynthetic pathway in the roots of black pepper. White and black columns refer to *P. nigrum* (susceptible) and *P. flaviflorum* (resistant), respectively. Three biological and technical replicates for each plant part were analyzed to ensure reproducibility and reliability. Error bars in the figures denote the standard error of expression levels from three biological replications. CK-8, CK-12, CK-24, CK-48, 8, 12, 24, and 48 on the x-axis refer to 8, 12, 24, and 48 h on control and 8, 12, 24, and 48 h after inoculation with *P. capsici*, respectively. The y-axis represents the relative expression level compared with ubiquitin. Each column represents the mean value plus SD (standard deviation) from three biological replicates. The different capital letters indicate significant differences at *P* = 0.05 level

To enhance the description of gene functions and the differences between susceptible and resistant black pepper species after inoculation with pathogen, qPCR analysis was performed to analyse the effects of *P. capsici* exposure on the roles of genes in the lignin synthesis and core phenylpropanoid pathways during the process of *P. capsici* interaction with the roots of black pepper lines. *PAL*, *C4H*, *CCR*, caffeoyl-CoA O-methyltransferase (*CCoAOMT*), and *4CL* were up-regulated in *P. flaviflorum* compared with *P. nigrum* after *P. capsici* inoculation (Fig. 6). Thus, the expression of phenylpropanoid pathway genes was analyzed in the root of wounding (injection of water) and *P. capsici* infection independently. In both black pepper plant roots, the *PAL* gene appeared to be stimulated at 8 h after inoculation and increased continuously. *PAL* activity was exhibited high activity in the resistant plants after inoculation for 48 h. However, a lower level of *PAL* activity was presented in the susceptible species after inoculated. Similarly, *C4H*, *CCR* and *CCoAOMT* were expressed in both the susceptible and resistant plants; their expression increased to a high level 24 h after pathogen inoculation in resistant plants, while only a slight increase in their expression was observed in the susceptible plants.

**Fig. 7:**
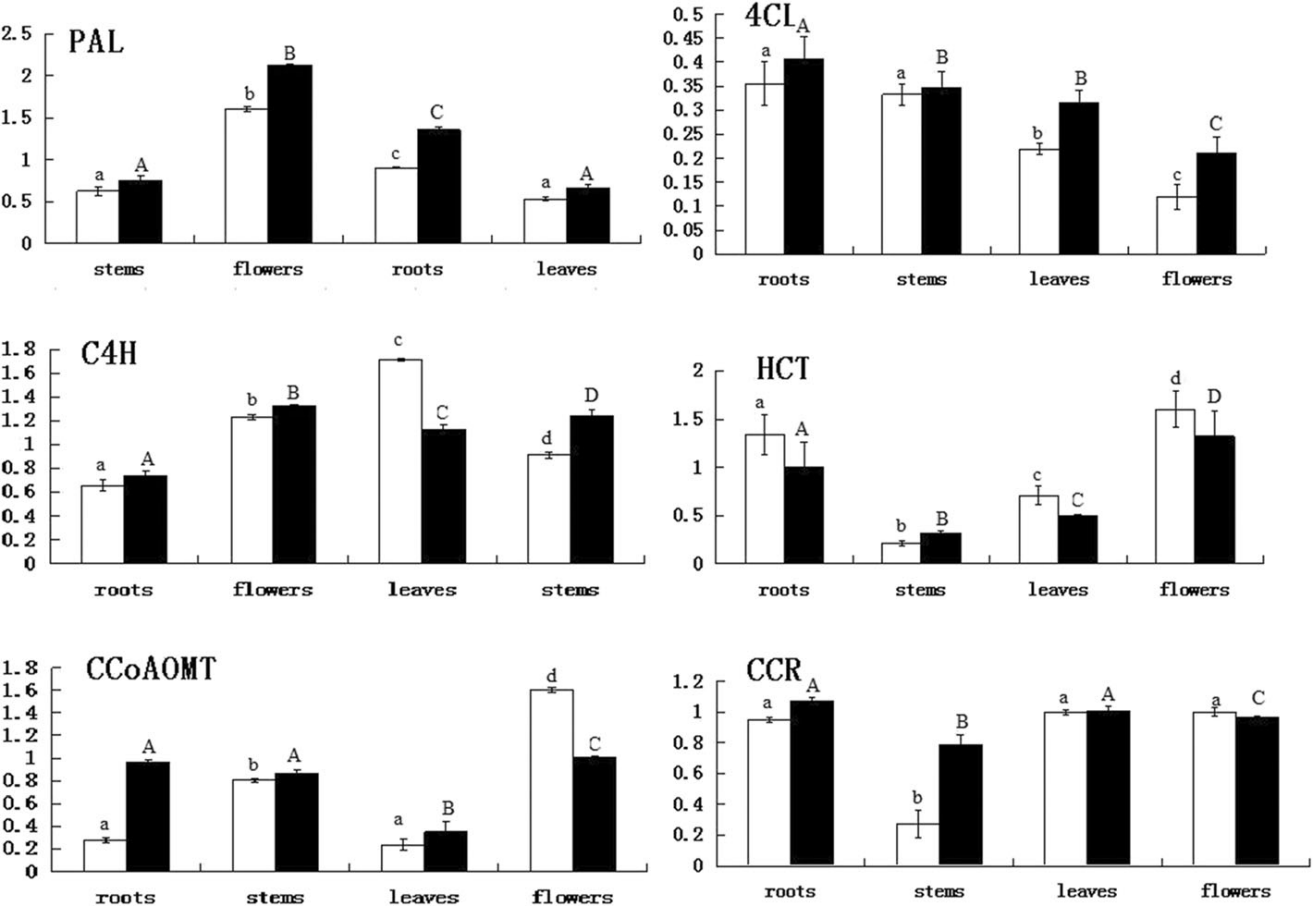
Expression of phenylpropanoid biosynthetic genes in different organs of black pepper as determined by qPCR. White and black columns refer to *P. nigrum* (susceptible) and *P. flaviflorum* (resistant), respectively. Three biological and technical replicates for each plant part were analyzed to ensure reproducibility and reliability. Error bars in the figures denote the standard error of expression levels from three biological replications. The y-axis represents the relative expression level compared with ubiquitin. Each column represents the mean value plus SD (standard deviation) from three biological replicates. The different capital letters indicate significant differences at *P* = 0.05 level

**Fig. 8:**
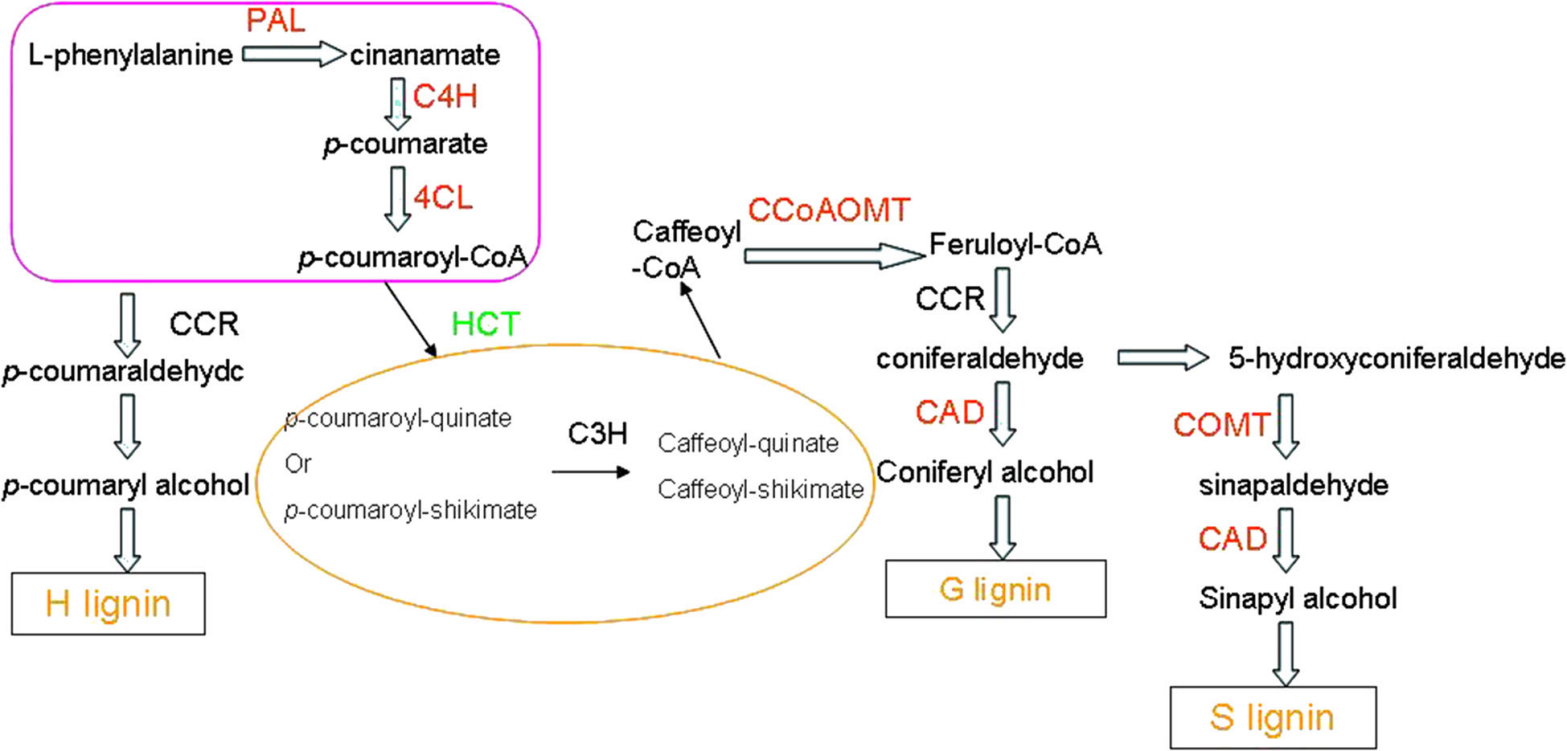
Overview of the putative Piper phenylpropanoid pathway and expression profiles of genes involved in this pathway [31]. The red box includes the critical enzymes that compose the entry pathway. The yellow route toward the production of monolignols is potential existence in this pathway. Enzymes shown in red or green indicate gene induction or suppression, respectively. Enzymes shown in black are those with both increased and decreased transcripts

qPCR analysis was used to determine phenylpropanoid biosynthetic gene expression in the leaves, flowers, roots and stems of the two species (Fig. 7). As the first enzyme in the phenylpropanoid biosynthetic pathway, *PAL* showed the maximum expression levels in flowers, roots and stems, whereas only moderate expression was observed in the leaves. The expression patterns of *4CL*, *CCoAOMT* and *CCR* were observably higher in the roots than in the stems and leaves, with the exception of *C4H*. Meanwhile *PAL*, *4CL*, *C4H*, *CCoAOMT* and *CCR* showed high expression levels in the resistant plants and slightly lower levels in the susceptible plants. Figure 8 have presented a summary of the expression profiles of the identified genes that are included in these pathways and the involvement of the phenylpropanoid pathway in the black pepper defence response against *P. capsici*.

### Measurement of enzyme activity

**Fig. 9:**
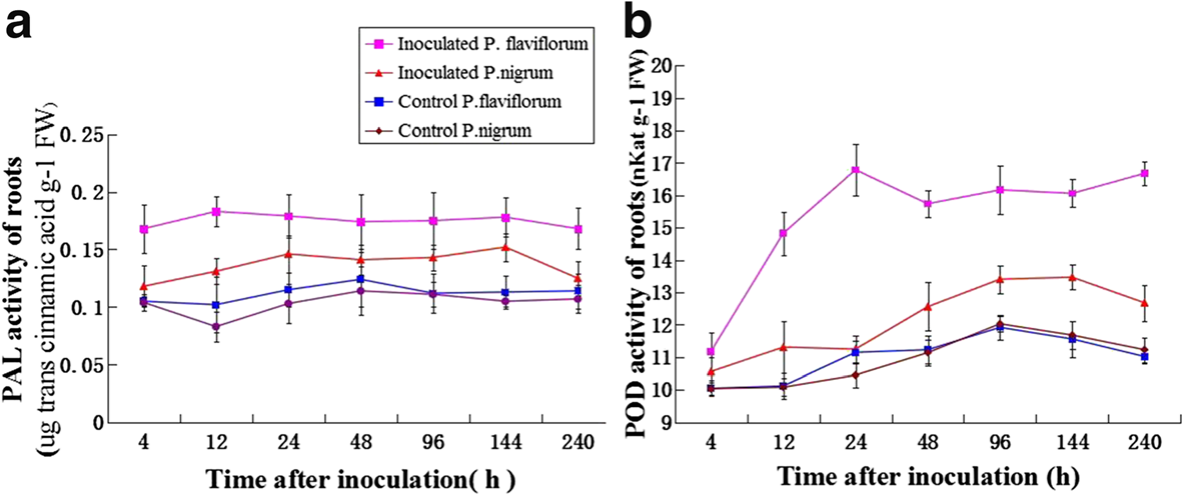
Enzyme activity in roots and stems of both control and inoculated susceptible and resistant black pepper plants at different time points after inoculation. **a** PAL, roots; **b** POD, roots. Error bars represent the SD for three independent experiments

PAL and POD, which were the main enzymes in the phenylpropanoid pathway, were measured in the susceptible and resistant species after *P. capsici* inoculation. Four hours after the roots of both black pepper plants were inoculated by *P. capsici*, PAL activity started to arise while 144 hours after being inoculated the activity declined. PAL activity levels remained high from 12–144 h after inoculation in resistant plants but remained relatively low in susceptible plants. Similar POD activity levels were observed in roots; these levels increased continuously in both two plants after pathogen inoculation and slightly increased. The results of enzyme activity analysis showed that the roots of resistant plants presented a higher level of POD and PAL activity than the susceptible plants (Fig. 9).

### Histochemical analysis of lignin localization

**Fig. 10:**
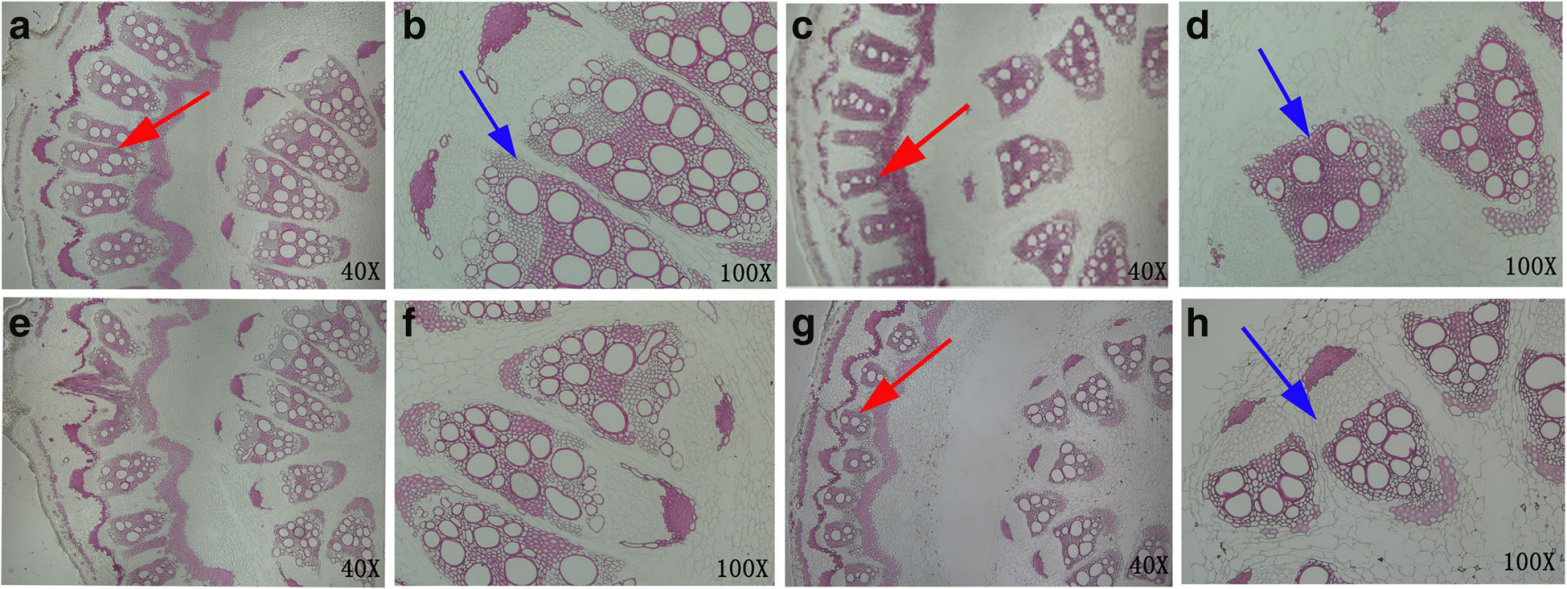
Histochemical analysis of lignin in stem cross-sections of control and *P. capsici*,-inoculated black pepper plants. Stem were stained with Wiesner reagents for detecting lignin. Pink staining with the Wiesner reagent indicates the presence of p-hydroxycinnamyl aldehyde end groups in lignin. **a**, **b**, **e**, **f** Sections from control and *P. capsici* –inoculated *P. flaviflorum* (resistant) stems. **c**, **d**, **g**, **h** Sections from control and *P. capsici* -inoculated *P. nigrum* (susceptible) stems. **a**, **b**, **c**, **d** Sections from control plants 10 d after treatment. **e**, **f**, **g**, **h** Sections from inoculated plants 10 d after treatment. Adjustments to magnification and illumination were made to allow optimal viewing of individual sections. **a**, **c**, **e**, **g**: 40×; **b**, **d**, **f**, **h**: 100×; Blue arrow showed xylem and numbers of lignified xylem bundles, vessels, and interfascicular fibres. Red arrow showed the screens and vessels in phloem

The phloroglucinol-HCl (Wiesner) reaction was used to detect different resistant and susceptible material changes in lignin and vascular structure before and after inoculation. A series of stages during stem growing could be reflected since both black pepper species were analysed before and ten days after being inoculated. A deep magenta in the Fig. 10 indicated that the site had a lignin deposition. The changes in phloem and xylem were observed between the control resistant and susceptible plants, including the increased number of screens and vessels in resistant (Fig. 10a–d, red and blue arrow showed). After the inoculation treatment, the vascular system was not much difference between control and inoculation in resistant plants (Fig. 10a, b, e, f). It was revealed by cross sections of inoculated susceptible stems that the xylem development was delayed and the amount of lignified inter-fascicular fibres, vessels, as well as xylem bundles was reduced (Fig. 10c, d, g, h. blue arrow showed). We speculated that the reduction in the number of catheter might also be one of the reasons of disease in black pepper.

## Discussion

Black pepper is an economically important crop, but foot rot caused by *P. capsici* has influenced its industry. The molecular mechanisms of disease resistance in black pepper remained unclear. Transcriptome sequencing, a significant tool, is being increasingly applied in the identification of the genes governing characteristic traits. Currently, NGS is being broadly employed for the qualitative and quantitative analysis of transcriptomes. Sheila et al. generated 71 million reads from stem-root tissue using the SOLiD platform (short reads) in black pepper [4]. The present study used transcriptome sequence analysis to obtain 147.44 Mb reads for *P. nigrum* and 147.49 Mb reads for *P. flaviflorum*, which corresponded to approximately 13.27G of raw sequence data, respectively. Subsequently, trinity was utilized to assembly raw reads for predicting unique genes. Among all the databases, including KEGG, NT, GO, Swiss-Prot and NR, annotated unigenes accounted for 68.46 % of the total amount of unigenes. This percentage was considered appropriate in view of the shortage of sequencing data regarding black pepper species. Similar percentages were found in bamboo and cucumber studies, which revealed 55 and 72 %, respectively, of the total number of unigenes [26, 27]. Moreover, high homology in the E-value distribution of the top hits in the NR database was observed, although no reference genome was available. Functional classification of black pepper genes identified genes associated with the “biological process”, “cellular component” and “molecular function” categories according to GO annotation. Genes annotated to “biological process” were identified only in cotton plants which were resistant to *V. dahliae* [28], these genes which participated in metabolic activity were also found in *Arabidopsis,* tobacco, alstroemeria, snapdragons as well as carnations which indicated that crucial metabolism happened in the process of resisting disease [29–31]. In the stage of cell proliferation while orchid was developing with stress resistance, gene categories related to “cellular component” were also detected. It was suggested that genes associated with membranes were altered and the membrane permeability changed accordingly which probably resulted from alterations in the constitution and construction of lipid bilayers [18]. Two DEG libraries were established to clarify the molecular mechanisms and regulation pathways of two species. It was revealed that in *P. flaviflorum* and *P. nigrum* 1,952 genes experienced significant down-regulation and 1,496 genes experienced significant up-regulation. The annotated unigenes were used to study primary and secondary metabolic pathways. KEGG pathway analysis could be conductive to clarifying physiological functions of genes through network [32, 33]. Further enrichment analysis of the two species noted that the phenylpropanoid pathway was significantly enriched in the groups with higher expression in *P. flaviflorum*.

Secondary metabolites, including terpenoid-, phenylpropanoid-, and nitrogen-containing substances that are derived from multiple pathways, play a fundamental role in a plant’s ability to fight against invading pathogens [34]. Systematic analyses of various genes in the phenylpropanoid pathway have been performed in *Arabidopsis*, tomato, rice and legume plants, including *Medicago truncatula*, alfalfa, pea and soybean [35–39]. These analyses indicated that this pathway was incorporated in the defence response [40]; while the entire variation of genes in phenylpropanoid pathway of black pepper has not been reported yet. In this study, the genes identified by RNA-Seq practically demonstrated the major branches of the phenylpropanoid pathway. These branches result in anthocyanin, flavonoid, lignin and proanthocyanidin synthesis; therefore, close attention was given to lignin synthesis and deposition. To determine the differences among these genes between *P. flaviflorum* and *P. nigrum* after being inoculated with *P. capsici*, qPCR was employed to analyse a subset of genes that played roles in lignin synthesis and the core phenylpropanoid pathway to obtain relative and absolute gene expression levels. After both black pepper plants were inoculated with *P. capsici*, the majority of examined genes were induced based on qPCR, and excluded *HCT* and *CCR*. Furthermore, lower expression levels and delayed increases in the genes were observed in the susceptible black pepper plants after inoculation. The results showed that these genes had a significant effect on the defence response of black pepper. In addition, inoculation with *P. capsici* induced a greater increase in POD enzymes in resistant cucumber, tobacco, and potato plants than in the susceptible plants [24, 25]. Moreover, it was observed in this study that after the resistant and susceptible pepper plants were inoculated by *P. capsici*, the activities of POD and PAL in both species were stimulated although the increasing rate and enzyme activity of susceptible plant were notably inferior to those of resistant plant. The interesting thing was that in both control plants the POD and PAL activities in roots arose which could be explained with the fact that the action of healing wounds in roots could enhance enzymes. This accorded with other investigations which reported that wounding stress was capable of inducing the expression of PAL and enhancing its activity [41].

Meanwhile only susceptible species were notably affected during developing with reduced amount of inter-fascicular fibres, vessels as well as lignified xylem bundles which could result in disease symptoms. It was discovered by Hwang [42] and Aguirreolea [43] that *P. capsici* infected pepper (*Capsicum annuum*) and its hyphae grew in cells of affected roots and stems in which the parenchyma cells were initially decayed.They both speculated that the increase in the flow resistance in the xylem was mainly caused by invasion of the dehydration symptoms. Similarly, black pepper infected by *P. capsici* exhibited xylem browning, blacking and other symptoms. Further evidences are needed to reveal the exact function of lignin in defending response to *P. capsici* of crops. Furthermore, phenylpropanoid products may have significant effects on defence response of plant, as not only signalling molecules, but phytoalexins. These genes were further described, which may offer candidates for improving black pepper’s gene. In addition, novel viewpoints regarding the plant disease resistance control might occur in case that the transcription factors having an effect on the phenylpropanoid pathway were identified.

## Conclusions

Illumina sequencing technology was applied to non-model species of the Piperaceae. Comparing susceptible and resistant black pepper lines by RNA-Seq, q-PCR of phenylpropanoid genes initial data were obtained which lead to a hypothesis that disease resistance is accompanied by elevated transcript levels of some phenylpropanoid genes in the resistent line, compared to the susceptible *P. nigrum*. Enzymatic assays of PAL and SOD currently support the hypothesis if a rise of putative defence genes, specifically in the resistant line. But the hypothesis needs to be confirmed in future studies by e.g. metabolomics of the root tissue at different time points. However, it is most likely that additional biosynthetic pathways and more complex signalling scenarios in the roots may be involved in this defence response.

## Methods

### Plant material and RNA extraction

The resistance of Black pepper germplasms to foot rot disease resulted from *P. capsici* was assessed and *P. flaviflorum* with resistance were screened using the protocol of Bhai [6]. One year old seedlings grown in Germplasm Repository of Black Pepper (Wanning, China) were cut off and used as materials. Each organs of mature plants which included flowers, leaves, stems as well as roots were collected from two distinct black pepper species. The roots of two non-infected species were collected for conducting Illumina sequencing. The materials for biological independent repeated trials of RNA sequencing were collected simultaneously. The potato dextrose agar plate was used to incubate *P. capsici* for one week which exhibited high aggressivity and could result in defoliation. The central of third internode from the tip of a rooted cutting was acupunctured with the sharp edge of a needle and a 3 mm inoculating disc taken from the growing margin of *P. capsici* was inserted into the acupuncture point. Subsequently a wet cotton pad was used to cover the inoculating point and prevent drying while a polythene strip was used to maintain the position of inoculating disc. The inoculated plants were incubated for eight, twelve, twenty-four, or forty-nine hours in a greenhouse with a constant temperature of 25–28 °C and a relative humidity of 75–90 %. The plants in the control group were treated and sampled using distilled water in the same way except that they were not inoculated by *P. capsici*. Every experiment was independently repeated for three times and the results were used for analysis. Root samples of all plants were collected at each sampling time point after each treatment for qRT-PCR. All samples were frozen in liquid nitrogen at once and then stored under the temperature of −80 °C and/or freeze-dried for the purpose of transcriptome analysing or extracting RNA. The samples were pulverized in liquid nitrogen by a mortar and RNeasy Plant Mini kit (Omega, USA) was used to extract total RNA. Two of them come from separate experiments and one is the same as that used in RNA-seq experiment about three biological replicates in qRT-PCR.

### Illumina sequencing

oligo (dT) beads were used to enrich poly (A) mRNAs in extracted total root RNA and fragmenting buffer was used to hydrolyse mRNAs into smaller pieces. The first strand cDNA was synthesized with reverse transcriptase as well as random hexamer primers and the second strand cDNA was synthesized with DNA polymerase I as well as RNase H (Invitrogen). Then a QiaQuick PCR extracting kit was applied to purify short fragments. Poly (A) tails were added into fragments to prepare the analysis and EB buffer was adopted to repair ends. Agarose gel electrophoresis was utilized to select proper short fragments for PCR amplifying and then sequencing adapters were connected to these fragments. The cDNA library was sequenced by Illumina HiSeq™ 2000 in Beijing Genomics Institute (BGI) genomic centre (Shenzhen, China) (http://www.genomics.cn/). The experiment for each sample was conducted in triplet.

### Read filtration and de novo assembly

Illumina HiSeq™ 2000 was used to produce data or raw reads which were then processed to obtain clean reads. Undesirable raw reads were removed according to following standard: (1) reads of low quality, that is, more than one fifth of which had a quality value which was not higher than ten; (2) sequences that unknown nucleotides took up five percent; (3) adaptors used for sequencing and reverse transcription. Trinity was used to assembly reads. First, the sequenced reads from 6 samples were combined and then RNA-seq data was assembled into contigs by Inchworm with the minimum K-mers coverage as well as default K-mer parameter of 3. Subsequently the reads were mapped back to contigs which were longest assembled sequences. Contigs could be detected with paired end reads from the identical transcript or the ranges between these them. Eventually non-Ns sequences that could not be extended from either end which could be defined as unique genes were obtained. All unigenes of multiple samples from the same species were assembled by TGICL to establish an individual set of unigenes without redundancy. Then the gene family clustering was conducted and the unique genes were divided into two classes. The similarity among the unigenes of one cluster was beyond seventy percent. The others were singletons with the prefix of Unigene. blastX was used to align unigene sequences against @blastdb and the e-value was lower than 0.00001. Sequence orientations were confirmed on the basis of the best hit in database. If the results generated from various databases exhibited non-conformity, @blastdb was given the first priority. Other sequences were provided as the assembler outputs. NR unigenes as long as possible were obtained through splicing sequences and removing redundancy of unigenes from each sample’s assembly with clustering software.

### Functional annotation

To make comparisons and perform alignments, unigene sequences were initially analysed using protein databases, such as NR, SWISS-PROT, KEGG, and COG, by applying BLASTX algorithms with a cut-off E value of 10^−5^. Moreover, unigenes were matched with Blastn to nucleotide (nt) databases (E-value ≤ 10^−5^) for protein identification. When the Illumina sequence direction could not be determined using the sequencing data directly, the direction was derived from BLAST annotations (ftp://ftp.ncbi.nih.gov/blast/executables/blast±/LATEST/). Regarding the nr annotation, GO terms were obtained by employing the Blast2GO program, to describe the biological processes and molecular function of cellular components [44]. To understand the gene function distribution, GO function categorization was performed using WEGO software [45] after GO annotations were available for all the unigenes. A number of unigenes could be clustered to the same KEGG pathway and to the same GO terms through the application of nt annotations in the KEGG pathway database [33]. The differential expression of genes were analysed through converting the read quantity of each contig in two samples into fragments per kilobase per million (FPKM). The expression abundance of each contig in analysed samples were calculated by the method based on MA plot with Random Sampling model (MARS) in DESeq package. Obtained network research was visualized as interacting proteins by means of interaction database which included all interactions in IntAct, BIOGRID, *Arabidopsis* PPI data curated from literature by TAIR curators, predicted interactome for *Arabidopsis* as well as *Arabidopsis thaliana* Protein Interactome Database. Cytoscape software (V2.6.2 http://www.cytoscape.org/) was used to perform network analyses.

### cDNA synthesis and real-time PCR

The RNA-Seq results were verified by conducting qRT-PCR on genes which exhibited upregulation and downregulation in both experimental conditions. Spectrophotometric analysis and 1 % agarose gel electrophoresis were adopted to assess the concentration and quality, respectively, of various total extracted RNAs after total RNA was extracted from various organs and treatments. First-strand cDNA synthesis was performed using 1 μg of high-quality total RNA to conduct reverse transcription (RT) (Toyobo Co. Ltd., Osaka, Japan). In total, 20 μL of the resulting cDNA was diluted 20-fold and then served as the template for real-time PCR. Primer 5.0 was used to design real-time PCR primers according to the sequences of *CCR*, *C4H*, *PAL*, *HCT*, *4CL* and *CCoAOMT* (Additional file 5: Table S1). By adopting a relative quantification approach, the calculation for the gene expression was performed in which the ubiquitin gene was considered the standard. Real-time PCR was performed in a 20 μL reaction mixture containing 10 μL of 1x SYBR Green Real-time PCR Master Mix (Toyobo, Osaka, Japan), 2 μL of template cDNA and 0.8 μL of each primer and DEPC-treated water. The reaction system was heated to 95 °C and denatured for five minutes, then the mixture was maintained at 95 °C for five seconds and DNAs were annealed and extended at 55 °C through 42 cycles. PCR was conducted with a CFX96 Real-Time system (Bio-Rad Laboratories, Hercules, CA, USA) and the products were analysed with Bio-Rad CFX Manager 2.0 software. The reliability and reproducibility were ensured through replicating biologically and technically for three times for each part of plant. The standard error in expression levels of three biological replications were denoted by error bars in the figures. The 2^-△△CT^ method [46] was used to quantify the relative expression levels of genes which were calculated as fold changes between selected each sample and the reference sample. The results were expressed as mean value ± SD. Duncan’s Multiple Range Test as well as ANOVA were used to conduct statistical assessment in qRT-PCR and *P* value was set to 0.05.

### Measurement of enzyme activities

Root samples were collected from black pepper plants which were treated with *P. capsici* or distilled water in a parallel way at the time point of 4, 12, 24, 48, 96, 144, and 240 hours. All samples were frozen in liquid nitrogen as soon as possible and then stored at −80 °C. Then 100 mg of each sample was ground with mortar in extraction buffer (sodium acetate 50 mM, pH 5.0). The extracting solutions were centrifuged at 14 000 g for 15 min under the temperature of 4 °C and the supernatants were collected as crude enzyme extractions used for determining enzyme activities. The method proposed by Dubery and Smit was used to determine PAL activity [47]. Guaiacol was used as the hydrogen donor at 470 nm to determine the activity of POD [48]. POD activity was expressed as nanokatal (nKat) per gram of sample.

### Histochemical test

After 10 d of treatment, the base stems of inoculated plants in two species were harvested to prepare freehand sections with the approximate thickness of 50–80 μm. Initially, the samples were fixed at 4 °C overnight in formaldehyde/acetic acid/alcohol (FAA) solution which contained 10 % (v/v) formaldehyde, 5 % (v/v) glacial acetic acid, as well as 50 % (v/v) ethanol. Wiesner reagent was used to conduct the lignin histo-chemistry tests [49] and Olympus fluorescence microscope (BX51, Olympus, Japan) was used to observe the sections directly.

### Availability of supporting data

The sequencing data in this study were stored in the NCBI Sequence Read Archive (SRA, http://www.ncbi.nlm.nih.gov/Traces/sra/) with the accession number of SRX708503.

## Additional files

Additional file 1: Data S1. The differentially expressed transcripts between *P. nigrum* and *P. flaviflorum*. (XLS 2838 kb)
Additional file 2: Figure S1. Comparison of the susceptible and resistant black pepper species transcriptomes with GO enrichment analysis. (A) Susceptible species and (B) resistant species.(The resistant and susceptible species showed differences transcript enrichment in genes involved in “phenylpropanoid metabolic process” and “cellular amino acid derivative metabolic process”). (PNG 2762 kb)
Additional file 3: Figure S2. Black pepper protein-protein interaction (PPI) network. Genes with high expression in *P. flaviflorum *are shown in red, and genes with high expression in PN are shown in blue. The node size correlates to the degree of difference in *P. nigrum/P. flaviflorum*. (PNG 2446 kb)
Additional file 4: Data S2. The differentially expressed transcripts enriched in phenylpropanoid metabolic pathways. (XLSX 13 kb)
Additional file 5: Table S1. Primers for qRT-PCR. (DOC 29 kb)

## Abbreviations

4CL: 4-coumarate CoA ligase
C4H: Cinnamate 4-hydroxylase
CAD: Cinnamyl alcohol dehydrogenase
CCoAOMT: Caffeoyl-CoA O-methyltransferase
CCR: Cinnamoyl-CoA reductase
COG: Clusters of orthologous group
EC: Enzyme commission
GO: Gene ontology
HCT: Hydroxycinnamoyl transferase
KEGG: Kyoto encyclopedia of genes and genomes
KOG: EuKaryotic orthologous group
NGS: Next-generation sequencing
PAL: Phenylalanine ammonia lyase
POD: Peroxidise
qRT-PCR: Quantitative real-time polymerase chain reaction
