# Supplementary figures and images for: De novo transcriptome sequencing of black pepper (Piper nigrum L.) and an analysis of genes involved in phenylpropanoid metabolism in response to Phytophthora capsici
Source: BMC Genomics. 2016 Oct 21;17:822. doi: 10.1186/s12864-016-3155-7 (PMC5075214; doi:10.1186/s12864-016-3155-7)

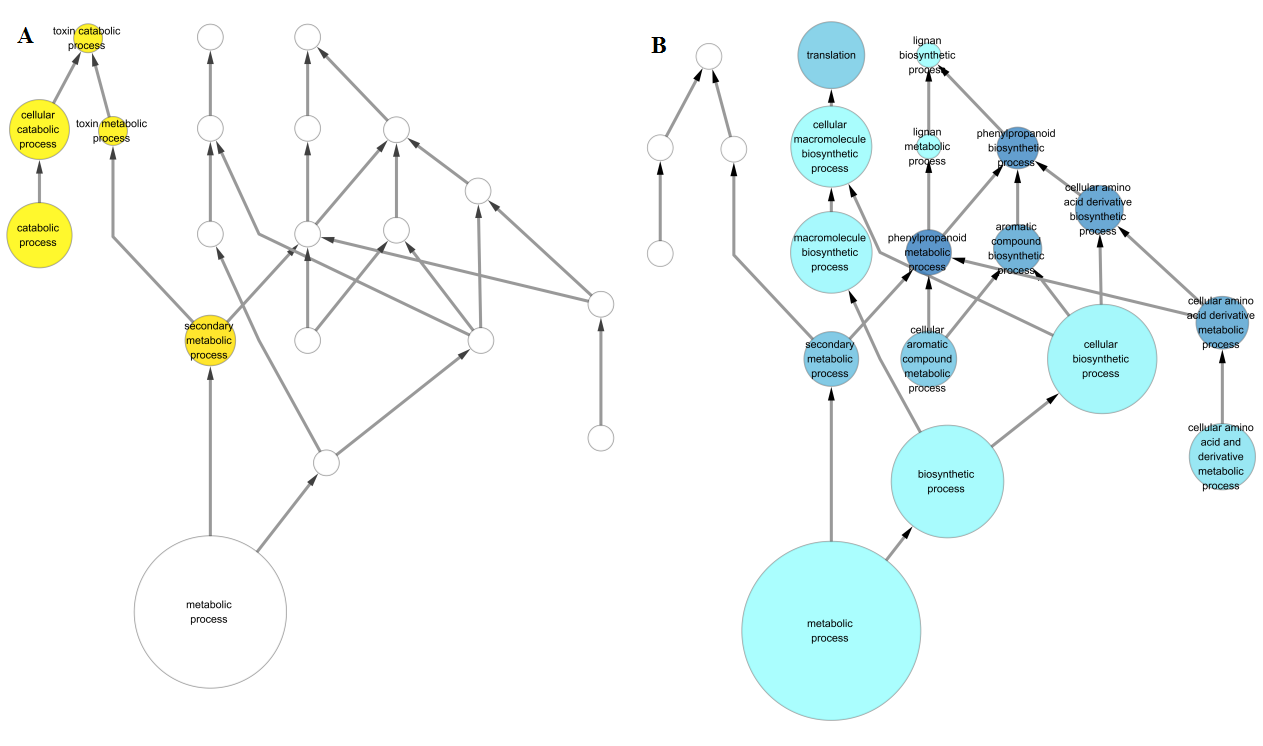

Supplement: Additional file 2: Figure S1. — Comparison of the susceptible and resistant black pepper species transcriptomes with GO enrichment analysis. (A) Susceptible species and (B) resistant species.(The resistant and susceptible species showed differences transcript enrichment in genes involved in “phenylpropanoid metabolic process” and “cellular amino acid derivative metabolic process”). (PNG 2762 kb) [file 12864_2016_3155_MOESM2_ESM.png]

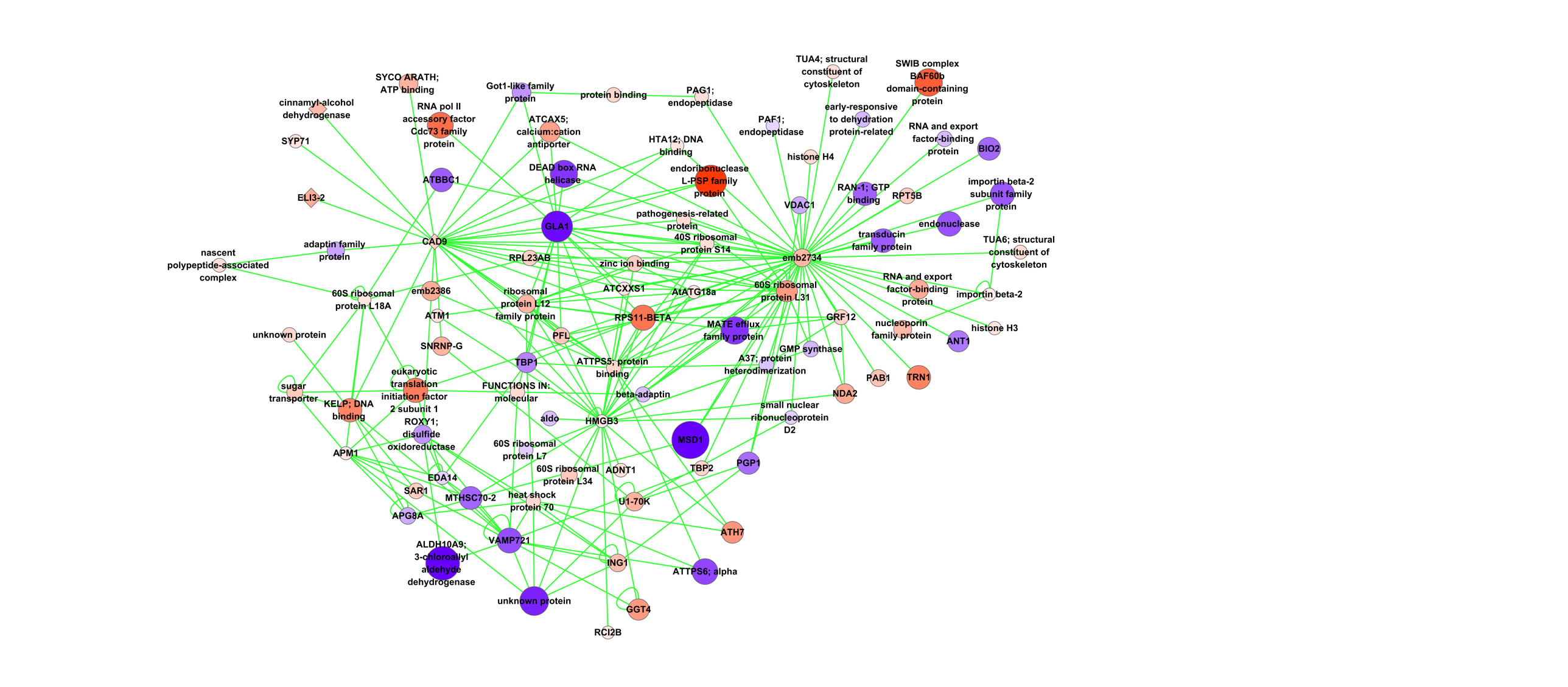

Supplement: Additional file 3: Figure S2. — Black pepper protein-protein interaction (PPI) network. Genes with high expression in P. flaviflorum are shown in red, and genes with high expression in PN are shown in blue. The node size correlates to the degree of difference in P. nigrum/P. flaviflorum. (PNG 2446 kb) [file 12864_2016_3155_MOESM3_ESM.png]
