# Supplementary material for: De novo transcriptome sequencing of black pepper (Piper nigrum L.) and an analysis of genes involved in phenylpropanoid metabolism in response to Phytophthora capsici
Source: BMC Genomics. 2016 Oct 21;17:822. doi: 10.1186/s12864-016-3155-7 (PMC5075214; doi:10.1186/s12864-016-3155-7)
Supplement: Additional file 5: Table S1. — Primers for qRT-PCR. (DOC 29 kb) [file 12864_2016_3155_MOESM5_ESM.doc]

| Additional data 3: Table S1 Primers for qRT-PCR. | | |
| --- | --- | --- |
| Description | F | R |
| phenylalanine ammonia-lyase | GCCGAAGCAGGACGAAGCCG | CTTTCCACCATTAGAGTTGC |
| 4-coumarate:CoA ligase | TCTGTCATCCCATAGCCC | CGATTCGGACGCTGAAGT |
| cinnamoyl CoA reductase | TGCACCGAGTTGGCGTAG | GGGTGGGAAGTGGCGAAGGAGAGAG |
| caffeoyl-CoA O-methyltransferase | CGGTTGAAGCATAGGTCC | CTCCGTTCTTCCTGAATTGGTA |
| cinnamate 4-hydroxylase | CTTGGAGGTTGTTGATAGG | CTAAGAAGATTTGCTACTTGTTCCC |
| ubiquitin | ACCATTACCCTGGAAGTTGAAAG | ACCATTACCCTGGAAGTTGAAAG |
| hydroxycinnamoyl transferase | GCCGCAGATTCTCAAGGA | CGAAGTCGCCGAAGTCAT |
